# Artificial neural network modeling of *p*-cresol photodegradation

**DOI:** 10.1186/1752-153X-7-96

**Published:** 2013-06-03

**Authors:** Yadollah Abdollahi, Azmi Zakaria, Mina Abbasiyannejad, Hamid Reza Fard Masoumi, Mansour Ghaffari Moghaddam, Khamirul Amin Matori, Hossein Jahangirian, Ashkan Keshavarzi

**Affiliations:** 1 Material Synthesis and Characterization Laboratory, Institute of Advanced Technology, Universiti Putra Malaysia, Serdang, Selangor 43400 UPM, Malaysia; 2 English Department, Faculty of Modern Languages and Communication, Universiti Putra Malaysia, Serdang, Selangor 43400 UPM, Malaysia; 3 Department of Chemistry, Faculty of Science, Universiti Putra Malaysia, Serdang, Selangor 43400 UPM, Malaysia; 4 Department of Chemistry, Faculty of Science, University of Zabol, Zabol, Iran; 5 Department of Chemical and Environmental Engineering, Faculty of Engineering, Universiti Putra Malaysia, Serdang, Selangor, 43400 UPM, Malaysia; 6 Otto-Schott-Institut, Jena University, Fraunhoferstr. 6, Jena, 07743, Germany

**Keywords:** Photodegradation, ANN-modeling, *p*-cresol, ZnO, UV-irradiation, Photocatalyst

## Abstract

**Background:**

The complexity of reactions and kinetic is the current problem of photodegradation processes. Recently, artificial neural networks have been widely used to solve the problems because of their reliable, robust, and salient characteristics in capturing the non-linear relationships between variables in complex systems. In this study, an artificial neural network was applied for modeling *p*-cresol photodegradation. To optimize the network, the independent variables including irradiation time, pH, photocatalyst amount and concentration of *p*-cresol were used as the input parameters, while the photodegradation% was selected as output. The photodegradation% was obtained from the performance of the experimental design of the variables under UV irradiation. The network was trained by Quick propagation (QP) and the other three algorithms as a model. To determine the number of hidden layer nodes in the model, the root mean squared error of testing set was minimized. After minimizing the error, the topologies of the algorithms were compared by coefficient of determination and absolute average deviation.

**Results:**

The comparison indicated that the Quick propagation algorithm had minimum root mean squared error, 1.3995, absolute average deviation, 3.0478, and maximum coefficient of determination, 0.9752, for the testing data set. The validation test results of the artificial neural network based on QP indicated that the root mean squared error was 4.11, absolute average deviation was 8.071 and the maximum coefficient of determination was 0.97.

**Conclusion:**

Artificial neural network based on Quick propagation algorithm with topology 4-10-1 gave the best performance in this study.

## Background

Environmental pollution on a global scale has drawn the attention of scientists to the vital need for friendly chemically clean processes. Phenolic compounds such as cresols are widely used in manufacturing products including cresol-based resin, herbicides, pharmaceuticals, surfactants and petrochemical [1]. *P*-cresol, water solubility above 21.5 g L^-1^ at 25°C, has been listed as a persistent priority, toxic chemical and the quantitative structure–activity relationship which indicates a significant threat to the environment [2-5]. Recently, advanced oxidation processes (AOPs) have been used as one of the practical technologies for the removal of the persistent pollutants [6-8]. Among the various AOPs, zinc oxide (ZnO) as heterogeneous photocatalysis showed great photodegradation due to its ability to destroy a wide range of the pollutants at ambient temperature and pressure, without generation of harmful by-products [5,9-13]. In the photodegradation process, the main effective operational parameters such as irradiation time, pH, photocatalyst amount and concentration of the pollutants were investigated under ultra violet (UV) and visible-light irradiation [13-17]. However, the complexities of the parameters behavior in the radiant energy balance, the spatial distribution of the absorbed radiation, mass transfer, and mechanisms of the photochemical degradation, cause misinterpretation of results. In addition, the kinetic of the photodegradation is quite difficult to determine [18]. The complexities have been big challenges for the traditional methods such as one-variable-at a time. The methods have been carried out by varying one parameter while fixing other variables constant. Since the variables are not completely independent of each other during the process, it might have adverse effects on the yield of the photodegradation [13,15]. On the other hand, the multivariate methods such as response surface methodology (RSM) consider the effect of the variables during the performance simultaneously, which could be a promising view [16]. However, the methods consider only two variables at a time, which could be a big disadvantage for the complex system with more than two variables. Furthermore, the method is involved with the complicated statistical calculation such as fitting process and regression analysis [17,19]. More recently, artificial neural networks (ANN) have been widely used for modeling chemical reaction processes [20-23]. The models possessed reliable, robust, and salient characteristics in capturing the non-linear relationships between variables in the complex system. Therefore, the successful photodegradation of many environmental organic pollutants such as ethylene-diamine-tetra-acetic acid [24], nitrogen oxides [25], nitrilotriacetic acid [26], 2,4-dihydroxybenzoic acid [27] and decolorization of CI Acid Blue 9 [28] was studied by the ANN. As observed, the ANNs simulated the behavior of the complex reaction system by using different algorithms such as Quick propagation (QP), Incremental backpropagation (IBP), Batch backpropagation (BBP) and Levenberg- Marquardt (LM) algorithm [20]. Among them, the backpropagation was a popular algorithm. Since the results of the photocatalytic processes under different conditions were estimated free of the complexities. In this work, the development of a multilayer feed-forward neural network model was used to predict the photodegradation% of *p*-cresol by ZnO under UV irradiation. To optimize the modeling, the results of the algorithms were compared by minimized root mean squared error (RMSE) and the percentage of absolute average deviation (AAD) while the coefficient of determination (R^2^) was maximized.

## Experiment

### Materials and methods

*P*-cresol (99.5%, fluka), NaOH (99% Merck), H_2_SO_4_ (95%–97%) and other required chemicals were of reagent grade, obtained from Merck and were used without further purification. The ZnO (99%, merck) has a surface area of 3.3 m^2^/g measured by static BET using thermo finnigan sorptomatic 1990 series analyzer. The particle size of ZnO recorded on nanophox facility was 0.4–0.5 μm. Band gap measured using PerkinElmer Lambda 35 UV/Vis/NIR was 3.02 eV. In all photocatalytic experiments, a litter of mixture ZnO with known quantities and *p*-cresol was irradiated for an appropriate time. Photocatalytic experiments were performed in a non-continuous mode (batch) binary reactor fitted with 6 W UV-A lamp [15]. The mixture was magnetically stirred (200 rpm) to maintain even distribution of suspension throughout the reactor and to eliminate mass gradient. Air was blown into the reaction solution using an air pump at a flow rate of 150 L/h to make the produced gas volatile (CO_2_), increase solution fluidization and finally to make oxygen accessible. Flowing cooled water into the binary cylinder kept the temperature at around 25°C. At specific time intervals, samples were drawn from the bulk solution. The samples were filtered through a 0.45 μm polytetrafluro-ethylene (PTFE) membrane. In order to compare the efficiency of the photocatalytic degradation of *p*-cresol, the filtrates were analyzed by UV-Visible spectrometry (Shimadzu, UV-1650pc) at the maximum absorption wavelength of *p*-cresol (277 nm). It should be mentioned that the small positive error of UV-Visible spectrometry in comparison with HPLC was ignored in this paper (results not shown). The percentage of photocatalytic degradation of *p*-cresol was calculated using Equation (1).

(1) $$\mathrm{Photodegradation}\%=\frac{\left(C_{0}-C\right)}{C_{0}}\times 100$$

where C_0_ = initial concentration of *p*-cresol, C = concentration of *p*-cresol after photo irradiation. All photocatalytic degradation experiments were carried out in duplicate. The initial photocatalytic degradation was investigated in the dark, in the absence of the photocatalyst and at normal pH (7.5). Results showed only 6% and 7% of *p*-cresol was photolysed and adsorbed by the UV irradiation and the photocatalyst surface respectively [29].

### Experimental design

The modeling of the photodegradation was carried out by NeuralPower software version 2.5 which is used in several researches [30,31]. To design the experiments, irradiation time, pH, amount of photocatalyst and *p*-cresol concentration were selected as independent variables (inputs); while photodegradation (%) was selected as the dependent variable (output). The design was performed in the laboratory to obtain the actual responses. The experimental values were then used for ANN modeling. The data were randomly divided into three sets as training, testing and validation data (Table 1) using the option available in the software. The training and testing data were used to compute and ensure robustness of the network parameters, respectively. The testing stage was also utilized to avoid over fitting by using control errors [32]. To assess the predictive ability of the generated model, validation data were considered [21]. The data consisted of six additional experiments which were in the range of values given for ANN modeling and excluded from training and testing (Table 1).

**Table 1:** **The independent variables as input, actual and predicated of *p*-cresol photodegradation as output for training, testing and validation set**

**Run**	**Irradiation time (min)**	**pH**	**Photocatalyst (g)**	***P*-cresol (ppm)**	**Photodegradation (%)**	
					**Actual**	**Predicted**
**Training Data**						
1	300	6	2	100	62.479	63.176
2	240	7.5	1.5	125	53.094	53.327
3	180	9	2	100	40.124	39.348
4	300	9	2	100	74.538	74.800
5	120	7.5	1.5	75	48.465	49.289
6	360	7.5	1.5	75	92.229	93.719
7	180	6	1	50	45.636	45.549
8	300	6	1	50	65.062	65.131
9	180	9	1	50	69.848	69.680
10	240	7.5	1.5	25	95.866	96.360
11	180	9	1	100	57.887	57.755
12	300	6	2	50	92.261	91.860
13	180	6	1	100	36.513	37.256
14	300	6	1	100	55.227	54.659
15	180	6	2	50	63.546	63.619
16	180	9	2	50	75.796	76.247
17	240	7.5	0.5	75	70.055	70.197
18	240	7.5	2.5	75	84.811	85.001
19	300	9	1	100	78.207	78.287
20	240	4.5	1.5	75	25.717	25.788
21	240	10.5	1.5	75	55.993	56.588
22	180	6	2	100	35.462	34.380
23	300	9	1	50	85.148	84.930
24	300	9	2	50	96.684	96.131
25	240	7.5	1.5	75	92.555	89.420
**Testing Data**						
26	150	7	1.5	75	58.750	58.462
27	210	8	1.5	50	85.330	91.185
28	240	7.5	1.5	50	98.000	96.208
29	180	7.49	1.5	75	68.802	71.687
30	240	7.5	1	75	92.083	88.658
31	270	8	1.5	75	88.967	90.029
**Validation Data**						
32	180	7	1.5	75	65.778	74.244
33	240	7.5	1.5	50	92.716	97.141
34	180	8	1.5	75	68.218	78.871
35	240	7.49	1	60	81.217	87.489
36	180	7.49	1.5	50	85.233	92.214
37	210	7.49	1.5	75	81.474	87.439
38	240	7.5	1.5	75	88.000	91.143

### The ANN description

Generally, ANNs are mathematical models, which consist of connected units (neurons/nodes) in different layers. The models are usually used to infer a function from observations of a particular process. The network consists of different layers which are inter-connected by parallel nodes. The nodes are simple artificial neurons, which mimic a biological neural network. The inter-connections of nodes are qualified by the associated weights. The layers include first layer (input), which sends data via the weights to the nodes of the second layer (hidden layer), and then to the third layer (output) [33]. Multi-layer perceptron is a class of networks, which consists of multiple layers with computational units. The units are usually interconnected in a non-directed cycle way such as feed-forward neural network. In the network, the hidden layers can be more than one layer, but a single hidden layer is universally suggested. The number of hidden nodes is obtained by trial and error calculation, which is examined from 1 to ‘n’ nodes. The inter-connection type of layers is multilayer normal feed-forward [33]. In the net, all the nodes of a particular layer are connected to all the nodes of the next layer. In addition, the inputs for hidden and output layers are calculated by performing a weighted summation of all the inputs received from the former layer. The weighted sum of the inputs is transferred to the hidden nodes, where it is transformed using a transfer function [34]. The output of hidden nodes in turn, acts as inputs to output nodes where it undergoes similar or different transformation. In this case, the ANN was trained by using the learning algorithmic program which included QP, IBP, BBP and LM algorithm [20] while the connection types were the multilayer feed-forward. The transfer function was the logarithmic sigmoid for both hidden and output layers [35]. Since the sigmoidal function is bounded between 0 and 1 the input and output data is normalized to the range by the software scaling. The scaled data are passed into the input layer, propagated to hidden layer to reach to the output layer of the network. The number of hidden node is obtained by trial and error calculation. The nodes as output layer firstly acts as a summing junction which combines and modifies the inputs from the previous layer using the equation (2) [24],

(2) $$y_{i}=\sum_{j=1}^{i}x_{i}w_{\mathrm{ij}}+b_{j}$$

where ‘y_i_’ is the net input to node ‘j’ in hidden or output layer, x_i_ are the inputs to node j (or outputs of previous layer). The ‘w_ij_’ is the weights which represents the strength of the connection between the ‘*i*^th^’ node and ‘*j*^th^’ node. The ‘*i*’ is the number of nodes and ‘b_j_’ is the bias associates with node ‘j’. Moreover, the learning rate and momentum coefficient for the networks are chosen by the software default values [33]. The results of the process are appeared as RMSE (Eq. 3) which is based on the difference between actual and predicted values (Table 1).

(3) $$\mathrm{RMSE}={\left(\frac{1}{n}\sum_{i=1}^{n}{\left(y_{i}-y_{\mathrm{di}}\right)}^{2}\right)}^{\frac{1}{2}}$$

where ‘n’ is the number of points, ‘y_i_’ is the predicted values and ‘y_di_’ is the actual values. The minimum RMSE of the examined nodes demonstrate the desirable networks. Furthermore, the performance of the ANN models was assessed on the basis of the coefficient of determination (R^2^) and the percentage of absolute average deviation (AAD) between the models predicted-actual values of the network. The R^2^ and AAD are calculated as equations (4 and 5),

(4) $$R^{2}=1-\frac{\sum_{i=1}^{n}{\left(y_{i}-y_{\mathrm{di}}\right)}^{2}}{\sum_{i=1}^{n}{\left(y_{\mathrm{di}}-y_{m}\right)}^{2}}$$

(5) $$\mathrm{AAD}=\left\{\left[\sum_{i=1}^{n}\left(\left|y_{i}-y_{\mathrm{di}}\right|/y_{\mathrm{di}}\right)\right]/n\right\}$$

where ‘n’ is the number of points, ‘y_i_’ is the predicted value, ‘y_di_’ is the actual value, and ‘y_m_’ is the average of the actual values. Therefore, the appropriate topologies are determined by minimum RMSE and ADD while the R^2^ is at maximum value.

## Results and discussion

### The ANN model training

**Figure 1:**
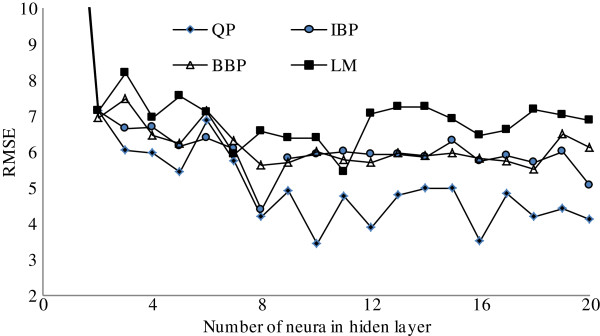
The performance of the network at different hidden neurons using, Incremental backpropagation algorithm (IBP), Batch backpropagation algorithm (BBP) and Quick propagation algorithm (QP).

In order to determine the optimum number of neurons in the hidden layer, a series of topologies was examined, in which the number of neurons was varied from 1 to 20. For instance, each topology was repeated 15 times to avoid random correlation due to the random initialization of the weights [36]. The RMSE was used as the error function. Moreover, the R^2^ and the AAD were used as a measure of the predictive ability of the network. Decision on the optimum topology was based on the minimum error of testing set values. These topologies have the lowest RMSE for the training and testing sets. Figure 1 illustrates the performance of the network for testing data versus of the number of neurons in the hidden layer using IBP, QP, BBP and LM algorithms. According to the RMSE the network with 8 hidden neurons produced the best performances when IBP algorithm was employed. Similarly, the best results were obtained with 10 hidden neurons using QP algorithm. The network with 18 hidden neurons produced the best results for BBP algorithm and best results obtained with 11 hidden neurons using LM algorithm. Therefore, the optimum topologies of the networks were 4-8-1, 4-10-1, 4-18-1 and 4-11-1 for IBP QP, BBP and LM algorithms respectively.

### Selection neural network model

Table 2 summarized the results of RMSE, R^2^ and AAD for the used algorithms in testing set. As shown, QP was at minimum of RMSE and AAD while, its R^2^ was at the highest value in comparison with the other algorithms. Therefore, the performance of QP with 4-10-1 topology was more effective than IBP, BBP and LM algorithms.

**Table 2:** **Statistical measures and performances of four learning algorithms on the photodegradation of *p*-cresol in suspension ZnO**

**Learning algorithm**	**The architecture**	**Testing data**		
		**RMSE**	**R**^**2**^	**AAD%**
Quick Propagation (QP)	4--10--1	1.399	0.975	3.048
Incremental Backpropagation (IBP)	4--8--1	1.792	0.965	4.184
Batch Backpropagation (BBP)	4--18--1	2.254	0.937	5.307
Levenberg-Marquardt (LM)	4--11--1	2.221	0.942	5.579

**Figure 2:**
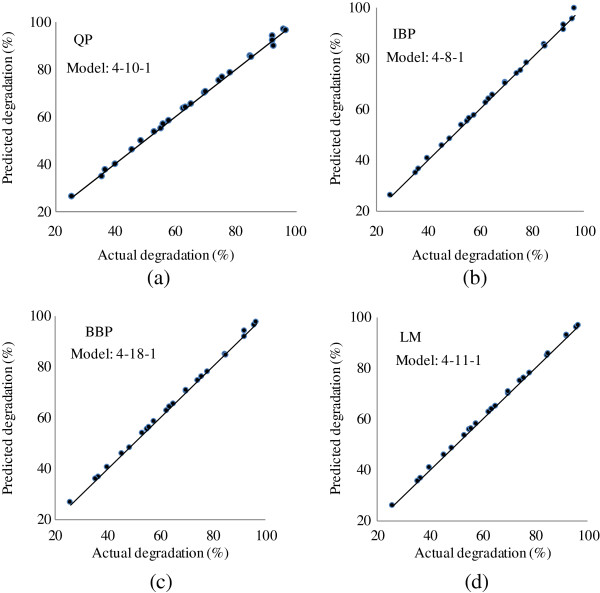
**The scatter plots of ANN predicted photodegradation % versus actual photodegradation % for training data set.** (**a**) Quick propagation (QP) algorithm, (**b**) Incremental backpropagation (IBP) algorithm, (**c**) Batch backpropagation (BBP) algorithm and (**d**) Levenberg- Marquardt (LM) backpropagation algorithm.

**Figure 3:**
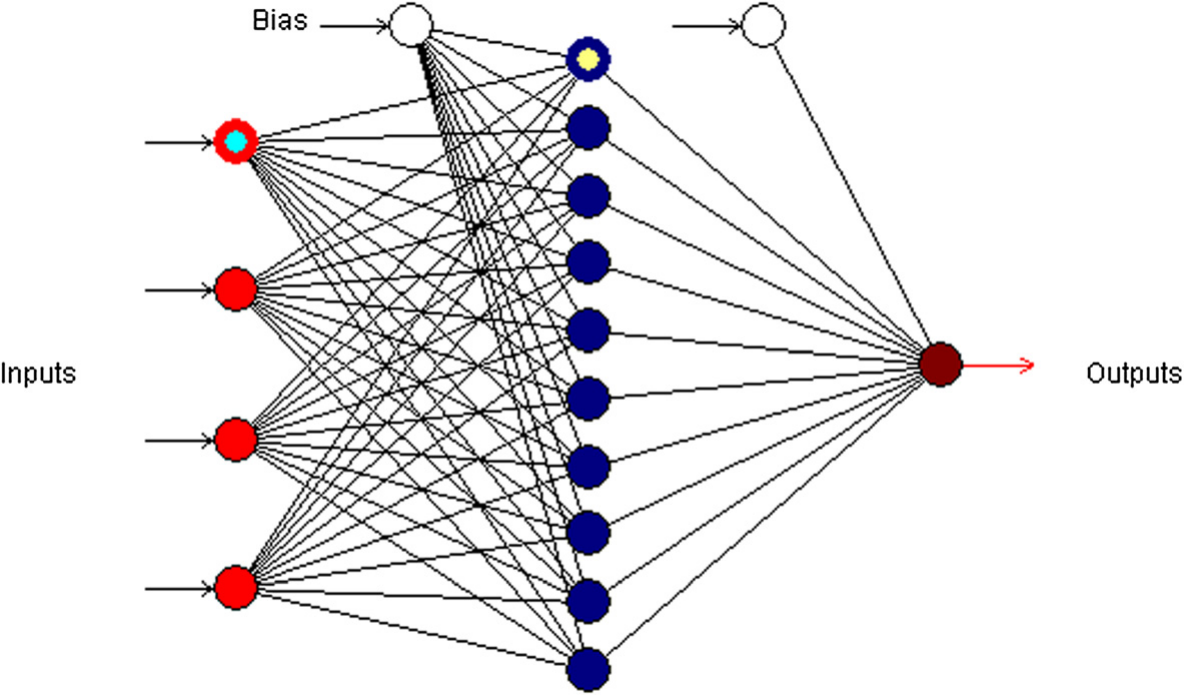
The multilayer feed-forward perceptron (MLP) network for quick propagation (QP) algorithm, the model consists of four inputs, one hidden layer with ten neurons and one output.

Table 1 presents the predicted values of the photodegradation for QP, IBB, BBP and LM algorithms. Figure 2 shows the predicted values versus the photodegradation actual values for the training set. As the comparison of the scatter plots demonstrates, the QP predicted model was well fitted to the actual values (R^2^ = 0.9997). Therefore, the QP with 4-10-1 topology (Figure 3) was considered as efficient model training for the photodegradation.

### Model validation

**Figure 4:**
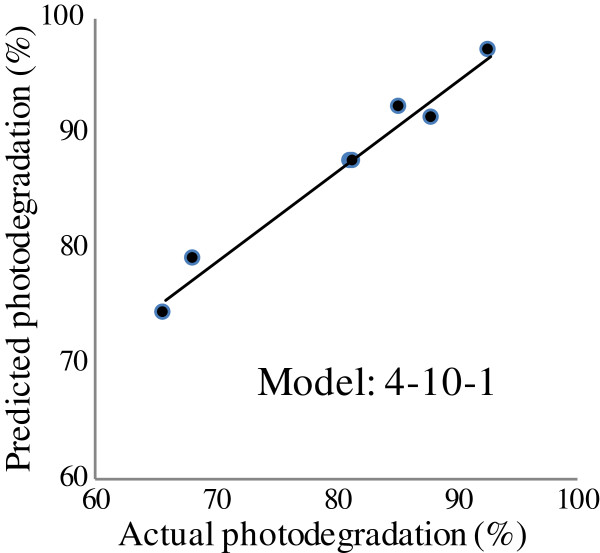
The scatter plot of ANN predicted photodegradation versus actual photodegradation for validation data.

The predictive ability of the generated model (4-10-1) was validated using a series of data which was excluded from training and testing data set (Table 1). Figure 4 presents the prediction versus actual of the photodegradation which was obtained in laboratory. As illustrated, the RMSE was 2.31; the R^2^ was 0.98 and the AAD% was 4.4 which indicated the great predictive accuracy of the model.

### The importance

**Figure 5:**
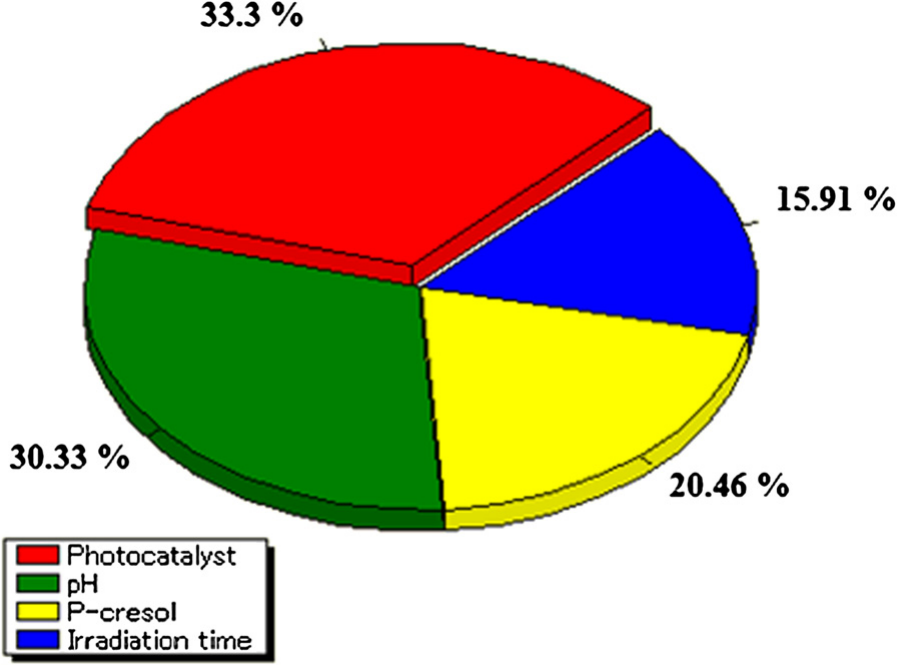
**Importance of effective parameters on percentage photodegradation of *p*-cresol.**

**Figure 6:**
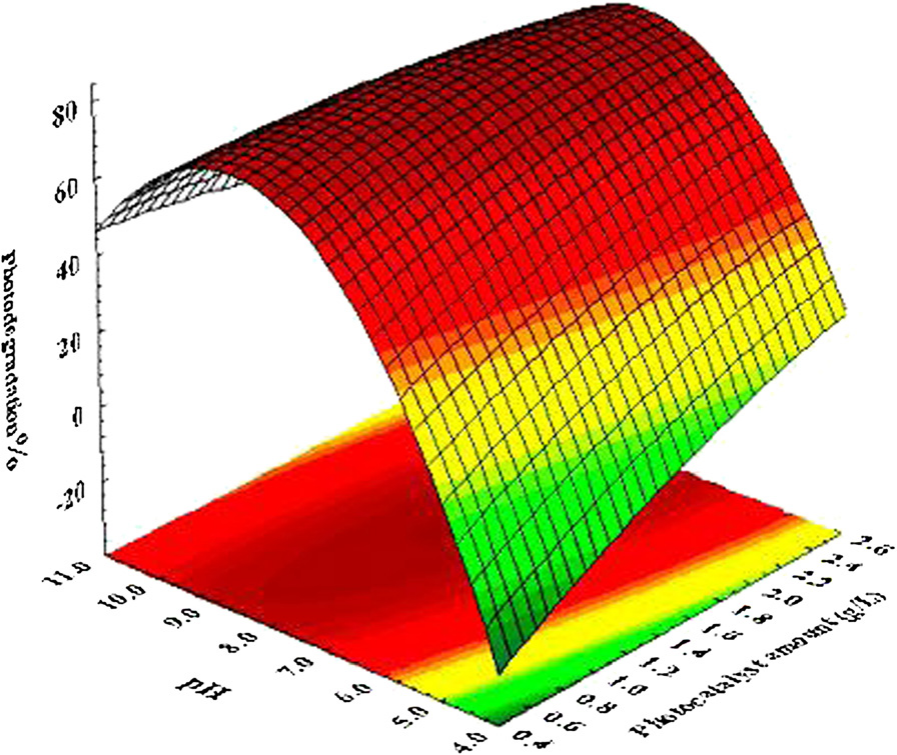
**Three dimensional plots of photocatalyst and pH effect on the photodegradation percentage.** The other variables were kept constant.

Figure 5 demonstrates the importance of effective parameters on the photodegradation as an output of the model. As shown the importance values of the parameters was photocatalyst amount > pH > p-cresol concentration > irradiation time in the selected range of the variables (Table 3). For further information, the optimum amounts of the importance factors, which were photocatalyst and pH, presented in Figure 6.

**Table 3:** Range and relative significance of the ANN input variables used in this work

**Input variables**	**Units**	**Range**	**Importance (%)**
Irradiation time	min	120-360	16.34
pH	-	4.5-10.5	30.31
Photocatalyst amount	g L^-1^	0.5-2.5	33.26
*p*-cresol concentration	mg L-1	25-125	20.09

## Conclusion

The modelling of *p*-cresol photodegradation was carried out by the ANN. The photodegradation was performed in ZnO suspension and under UV irradiation. The model contained input, hidden and output layers. The inputs included irradiation time, pH, photocatalyst amount and concentration of *p*-cresol while the photodegradation % was the output. To obtain the optimum model, ANN was trained by QP, IBP, BBP and LM algorithms. The minimum RMSE values through the repeating data were used as indicator to determine the number of nodes in the hidden layer for each algorithm. According to the minimum RMSE, the 4-10-1, 4-8-1, 4-18-1 and 4-11-1 topologies were selected for the algorithms. To compare the optimized topologies, the RMSE was used as the error function; R^2^ and ADD employed as an index of the network predictive ability. The comparison of the algorithm indicated that the QP had minimum RMSE, 1.3995, AAD%, 3.05, and maximum R^2^, 0.98, for the testing set. Furthermore, the results of QP validation were 0.97, 2.3 and 4.4 for R^2^, RMSE and AAD% respectively. In conclusion, the QP gave the best performances and was selected as the process’ model.

## Competing interests

Are there any non-financial competing interests (political, personal, religious, ideological, academic, intellectual, commercial or any other) to declare in relation to this manuscript? The authors declare that they have no competing interests.

## Authors’ contributions

YA (AB, JY, MT, ES) AZ (FG) MA (MT, FG), HRFM (ES), MGM (ES), KAM (FG) HJ (FG), AK (MT). AB carried out the catalyst design and ligand screening studies. JY carried out the synthesis, purification and characterization of the compounds. MT carried out the computational experiments. FG conceived of the study, and participated in its design and coordination and helped to draft the manuscript. All authors read and approved the final manuscript.
